# Equine Parvovirus-Hepatitis Frequently Detectable in Commercial Equine Serum Pools

**DOI:** 10.3390/v11050461

**Published:** 2019-05-21

**Authors:** Toni Luise Meister, Birthe Tegtmeyer, Alexander Postel, Jessika-M.V. Cavalleri, Daniel Todt, Alexander Stang, Eike Steinmann

**Affiliations:** 1 Department of Molecular and Medical Virology, Faculty of Medicine, Ruhr-University Bochum, 44801 Bochum, Germany; Toni.meister@rub.de (T.L.M.); Daniel.todt@rub.de (D.T.); Alexander.stang@rub.de (A.S.); 2 Institute for Experimental Virology, TWINCORE Centre for Experimental and Clinical Infection Research, a joint venture between the Medical School Hannover (MHH) and the Helmholtz Centre for Infection Research (HZI), 30625 Hannover, Germany; birthe.tegtmeyer@twincore.de; 3 University of Veterinary Medicine Hannover, Institute of Virology, 30559 Hannover, Germany; alexander.postel@tiho-hannover.de; 4 Department for Companion Animals and Horses, University of Veterinary Medicine, 1210 Vienna, Austria; Jessika.Cavalleri@vetmeduni.ac.at

**Keywords:** equine parvovirus-hepatitis, horses, commercial horse serum, phylogeny

## Abstract

An equine parvovirus-hepatitis (EqPV-H) has been recently identified in association with equine serum hepatitis, also known as Theiler’s disease. This disease was first described by Arnold Theiler in 1918 and is often observed after applications with blood products in equines. So far, the virus has only been described in the USA and China. In this study, we evaluated the presence of EqPV-H in several commercial serum samples to assess the potential risk of virus transmission by equine serum-based products for medical and research applications. In 11 out of 18 commercial serum samples, EqPV-H DNA was detectable with a viral load up to 10^5^ copies/mL. The same serum batches as well as three additional samples were also positive for antibodies against the EqPV-H VP1 protein. The countries of origin with detectable viral genomes included the USA, Canada, New Zealand, Italy, and Germany, suggesting a worldwide distribution of EqPV-H. Phylogenetic analysis of the EqPV-H NS1 sequence in commercial serum samples revealed high similarities in viral sequences from different geographical areas. As horse sera are commonly used for the production of anti-sera, which are included in human and veterinary medical products, these results implicate the requirement for diagnostic tests to prevent EqPV-H transmission.

## 1. Introduction

Parvoviruses are small, non-enveloped viruses with a DNA genome typically encoding at least two major gene complexes with the non-structural protein (NS1, multidomain nuclear phosphoprotein) and capsid protein (VP1). In recent years, many viruses have been newly identified or reassigned to the family *Parvoviridae*, which is divided into two subfamilies, the *Densovirinae* and the *Parvovirinae*, according to whether they infect invertebrates or vertebrates, respectively [1,2]. Both subfamilies are further divided into various genera based on their genome organization.

The parvovirus subfamily *Parvovirinae* is a large family with a wide host range, divided into eight genera that can infect humans, domestic animals, and wild animals [1,3,4].

Recently, a novel equine parvovirus (equine parvovirus-hepatitis, EqPV-H) was discovered in the serum of a horse that died of Theiler’s disease indicating that EqPV-H could be the causative agent of Theiler’s disease and also of subclinical infections [5,6,7]. Theiler’s disease is also known as equine serum hepatitis and was initially described by Sir Arnold Theiler in 1918 [8]. After experimental vaccination studies to prevent African horse sickness, Theiler observed hundreds of cases of a highly fatal form of hepatitis. Theiler’s disease or serum hepatitis has since been reported worldwide after treatment with a variety of equine serum products, for instance tetanus antitoxin, botulinum antitoxin, antiserum against Streptococcus equi as well as pregnant mare’s serum, and equine plasma [5,8,9,10,11,12]. The clinical disease among horses receiving these products has a high fatality rate, and the incidence of fulminant hepatitis in these outbreaks was in the range of 1.4%–18% [13]. Recent studies provided evidence for the association of EqPV-H and Theiler’s disease. A prospective study on Theiler’s disease cases described the detection of EqPV-H in 18 consecutive cases of Theiler’s disease that occurred after administration of an equine-based biological product [13]. The same authors also reported EqPV-H infection in 9 out of 10 cases of Theiler’s disease in the absence of an equine biological product administration and noted 54% virus positivity of tested in-contact horses [14].

Given the potential risk of EqPV-H contaminated equine serum products for medical as well as research applications, in this study we investigated the prevalence of EqPV-H among commercial equine serum pools from various countries worldwide. To this end, a total of 18 serum samples from different providers were analyzed for the presence of anti-EqPV-H-VP1-antibodies and EqPV-H DNA. The results indicate that EqPV-H is highly prevalent in commercial horse serum around the world and that blood-based products derived from equine donors should be tested for EqPV-H. 

## 2. Materials and Methods

### 2.1. Serum Sample Collection

A total of 18 different horse serum samples were collected from a variety of providers. The samples were shipped and stored at −20 °C until further analysis. Thaw and freeze cycles were kept at a minimum. 

### 2.2. Detection of EqPV-H DNA

Viral DNA was extracted with a viral DNA Kit from Qiagen (Cat. No. 1048147, Hilden, Germany) according to the manufacturer’s recommendations. DNA samples were stored at −20 °C until further analysis. A probe-based quantitative real-time polymerase chain reaction (qRT-PCR) was used with primers and probe designed and provided by Dr. Amit Kapoor as described before [5]. A serial dilution of a plasmid containing the EqPV-H VP1 sequence was generated as standard row for the quantification of EqPV-H within the samples tested. Fluorescence was assessed with a LightCycler 480 (Roche, Mannheim, Germany).

### 2.3. Detection of Anti-EqPV-H Antibodies

Samples were analyzed regarding the presence of anti-EqPV-H-VP1 antibodies using the luciferase immunoprecipitation system (LIPS) as described by Burbelo et al. [15,16] and Pfaender et al. [17]. For the EqPV-H-LIPS, the antigen VP1 was produced as described by Divers et al. [5]. Relative light units (RLU) were measured in a plate luminometer (LB 960 XS3; Berthold, Bad Wildbad, Germany). For calculation of sensitivity, a cut-off limit, analogous to Burbelo et al. (2012) and Pfaender et al. (2015), was determined and defined as the mean RLU plus 3 standard deviations (SD) of a EqPV-H negative horse serum. A cross-reaction of the LIPS with other related parvoviruses cannot be excluded.

### 2.4. Sequencing and Phylogeny

For sequence analysis, two PCRs (I and II) were designed within the NS1 of EqPV-H (Table 1 and Figure 3A). PCR was performed using the Expand High Fidelity PCR System (Roche Diagnostics) in a total volume of 50 µL containing 5 µL of purified DNA, 5 µL of 10× buffer, 200 µM of each dNTP, 10 pmol of each primer, and 0.375 µL of Taq Polymerase. 

The PCR profile was the following: 95 °C for 2 min, 45 cycles of 95 °C for 20 s, 60 °C for 30 s, and 72 °C for 1 min 30 s, followed by a final extension at 72 °C for 10 min. PCR products were visualized on a 2% agarose gel, excised, and purified using a Monarch^®^ DNA Gel Extraction Kit (New England Biolabs). Purified products were then sent for Sanger sequencing using the applicable PCR primers.

Phylogenetic trees were constructed using the maximum likelihood method and general time reversible model [18,19] implemented in MEGA X version 10.0.5 [20]. The tree is drawn to scale, with branch lengths measured in the number of substitutions per site.

### 2.5. Purification of Viral Particles

Particle associated nucleic acid (PAN) purification was performed in detail as originally described [21]: 11 mL of horse serum was clarified (3220 g, 30 min) and subsequently filtered through a 0.22 µm pore-size sterile filter to eliminate particles of higher density and mass such as bacteria, eukaryotic cells, or fragments of them. To concentrate virus particles and separate them from particles of lower density, 10 mL of sterile filtered serum was layered onto 2 mL of 30% (*wt/vol*) sucrose in PBS, followed by ultracentrifugation for 3 h in an SW41 rotor at 30,000 rpm. For preparation of DNA, the pellet was resuspended in 250 µL of PBS containing 20 mM MgCl_2_. To degrade DNA that is not inside a particle, a DNase step was performed by the addition of 5 U of DNase I followed by incubation at 37 °C for 30 min, followed in turn by DNA extraction with a Blood-Mini-Kit (Qiagen).

## 3. Results and Discussion

### 3.1. EqPV-H DNA is Frequently Detectable in Commercial Horse Sera

To evaluate the potential presence of EqPV-H in commercial serum pools, eighteen different serum samples were obtained from different countries with a specific serum type and number of individual horses per pool (Table 2).

**Figure 1:**
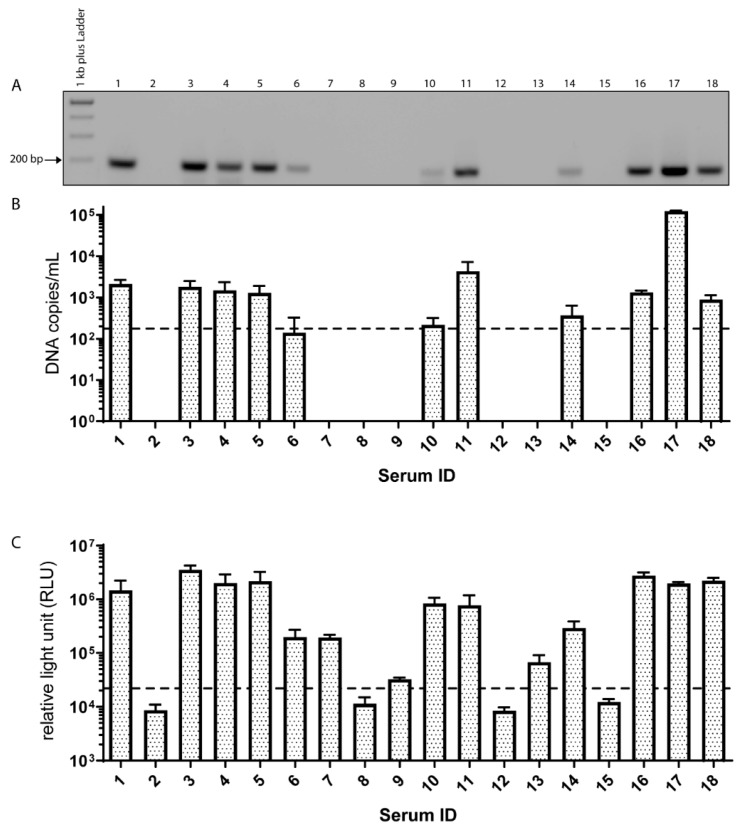
Occurrence of EqPV-H DNA and antibodies within commercial serum samples. (**A**) Agarose gel electrophoresis of quantitative PCR of commercial serum pools. (**B**) Viral loads of EqPV-H were determined using qPCR and are displayed in DNA copies/mL. The dotted line indicates the limit of detection. Every bar represents the mean of 5 technical replicates performed in 3 independent measurements. (**C**) Depicted are anti-EqPV-H antibodies as RLU measured in triplicates. The cut-off was determined by the mean value of the EqPV-H negative serum plus 3*SD and is indicated by a dashed line.

Eleven out of 18 commercially available sera tested positive for EqPV-H DNA, illustrated using gel electrophoresis of the qPCR products (Figure 1A). As depicted in Figure 1B, quantification of the viral DNA revealed loads ranging from 10^2^ to 10^5^ DNA copies/mL (Figure 1B). Next, the commercial horse serum samples were tested for the presence of anti-VP1-antibodies via LIPS assay. All EqPV-H DNA positive samples also yielded positive results in the antibody assay, while three additional sera were exclusively anti-VP1-antibody positive (7, 9, and 13) (Figure 1C). However, the measured relative light units (RLU) of these three seropositive samples were lower compared to the eleven sera that had also been tested DNA positive (Figure 1C). Due to the pooling of sera from several horses for one commercial serum batch, a potential dilution has to be considered, and the detection limit of EqPV-H PCR was determined at 175 DNA copies/mL. In line with these results, tetanus-antitoxins as one of the most common medical products administered to horses tested positive for EqPV-H in several cases of Theiler’s disease [13]. Importantly, an experimental infection study had previously confirmed transmission of EqPV-H from a PCR-positive biological product to seronegative horses [5]. To evaluate, if the detected parvovirus DNA was encapsidated or just plain unprotected DNA, we performed PAN DNA isolation [21] with serum 1 as one example. This method contains a DNAse treatment step, which degrades all unprotected DNA, thus selects highly specific for DNA protected by a capsid. After purification we performed quantitative PCR for both the DNA isolation with and without selection for PAN-DNA. We found that around 1% of total parvovirus DNA could be recovered using this method. A recovery rate of 1% corresponds to our own findings with various viruses (DNA, RNA, enveloped, and non-enveloped), spiked to PBS for evaluation of this method. The relatively high losses can be either explained by the high stringency of the method or simply by presence of non-protected nucleic acid naturally occurring for different viruses. Either way, for this horse serum it is highly likely that parvovirus DNA is encapsidated in viral particles and so viral particles are present. Due to the lack of an infection cell culture system, we could not determine if these particles are infectious.

Of note, serum samples 8 to 13 have also been tested previously by us for the presence of equine hepacivirus (EqHV), equine pegivirus (EPgV), and Theiler’s disease associated virus (TDAV) [22]. All samples were shown to be EPgV and EqHV positive except serum sample 12, which tested negative for EqHV. Furthermore, TDAV was detected in samples 10, 11, and 13 [22]. In summary, these results demonstrate a frequent detection of EqPV-H in commercial serum pools with 61.10% PCR positivity and 77.70% sero-positivity.

### 3.2. EqPV-H Can be Detected in Different Countries Worldwide

**Figure 2:**
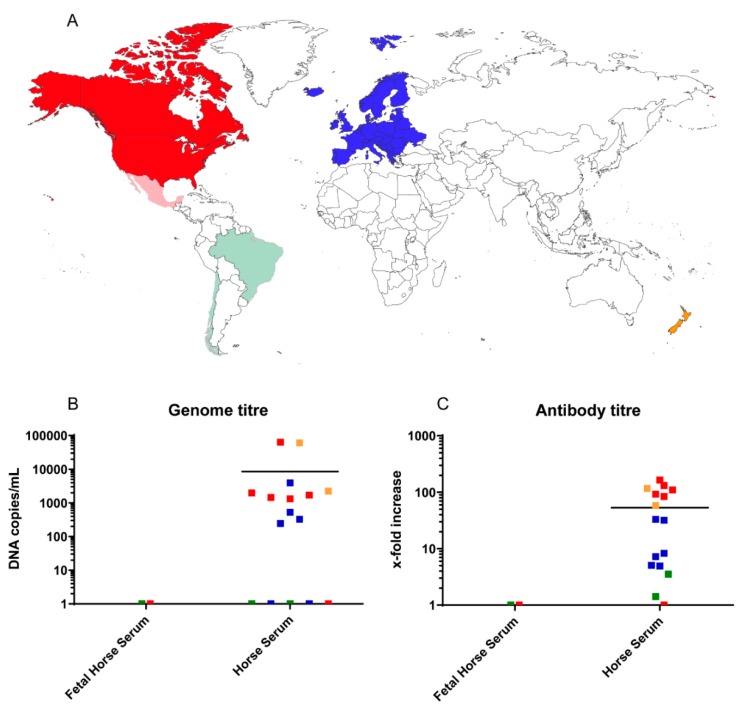
The origin of the serum pools is indicated on a world map (**A**). Reduced opacity indicates EqPV-H negative serum pools. Graphs (**B**) and (**C**) show the correlation of age (determined by the serum type) and origin on EqPV DNA and antibody prevalence, respectively. Every dot represents the mean of 5 technical replicates performed in two independent measurements. Negative samples were assigned the value 1. Red color corresponds to North America, while sera from South American countries are colored green. Blue indicates serum samples originating from Europe and orange belong to samples from Oceania (A–C).

As the novel parvovirus has only been recently described [5], prevalence studies with EqPV-H have so far only been performed in the USA and China. Lu et al. showed that EqPV-H has a very low genetic diversity between farms in the same geographic region, similar to what has been observed in the USA [5,7]. When comparing the origin of the here-tested commercial sera regarding the presence of EqPV-H DNA and antibodies, all South American sera (green) tested negative for EqPV-H, while the majority of sera from North America (red), Europe (blue), and Oceania (orange) were positive for EqPV-H DNA and antibodies (Figure 2A). These included the countries New Zealand, the USA, Italy, Germany, and Canada indicating a worldwide circulation of EqPV-H. The two fetal horse sera that were included in the study tested EqPV-H PCR and antibody negative in contrast to the adult horse sera (Figure 2B,C). However, further investigations with more samples are needed to determine the importance of EqPV-H for vertical transmission and prevalence in young horses. However, PCR- and sero-negativity could also be a coincidence due to the origin of the fetal horse serum from Central and South America.

### 3.3. Sequence and Phylogenetic Analysis of EqPV-H in Commercial Serum Pools

**Figure 3:**
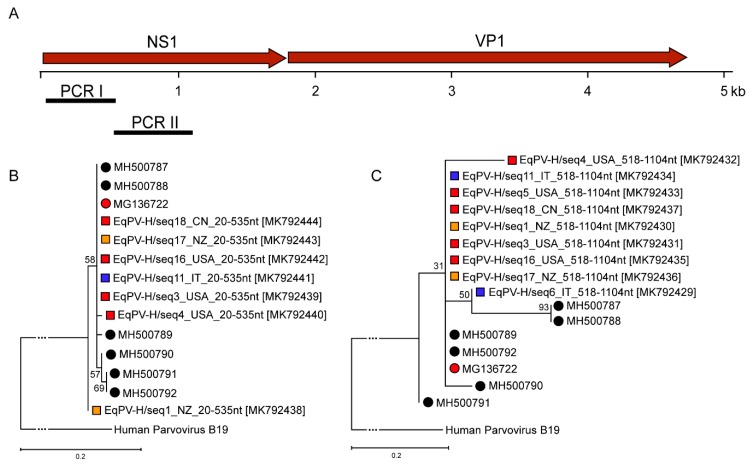
(**A**) Genomic organization of EqPV-H and primer positioning for PCR I-II. (**B**) Phylogenetic tree based on the fragments generated using PCR I. (**C**) Phylogenetic tree based on the fragments generated using PCR II. Evolutionary history was inferred by using the maximum likelihood method and general time reversible model. The trees with the highest log likelihood are shown. Red corresponds to North America, while blue indicates serum samples originating from Europe and orange matches samples from Oceania. Black corresponds to Asian strains. Circles indicate previously published EqPV-H sequences identified by various groups, whereas squares label newly found EqPV-H sequences within this study. Human parvovirus B19 was used as outgroup to root the tree.

For a molecular characterization of the EqPV-H positive samples, primers (Table 1) in the NS1 gene were designed to obtain two PCR fragments (Figure 3A). Due to low viral loads being present for only some of the qPCR positive samples, sequencing of PCRI and II fragments was possible. We were able to recover 7/11 and 9/11 sequences for PCRI and II, respectively. These sequences were submitted to the GenBank database with the accession numbers indicated in Table 3. A maximum likelihood approach was used, and robustness of the trees tested with a bootstrap analysis was performed with a replicate rate of 1000. As depicted in Figure 3B,C, the obtained sequences were highly similar for all commercial serum pools around the world and clustered with the published American and Asian EqPV-H sequences (Figure 3B,C). This can be observed for two individually amplified regions in the NS1 gene. These results point to a high conservation between the world-wide circulating strains and low genetic variability of the EqPV-H strain.

## 4. Conclusions

In this study, we investigated the occurrence of EqPV-H DNA and antibodies within commercial horse sera. We showed that anti-EqPV-H DNA and EqPV-H antibodies are frequently detectable in commercially available horse sera from various origins indicating a worldwide circulation of EqPV-H infections. As horse sera are commonly used for production of anti-sera, which are licensed for various treatments in different animal species as well as humans (snake antivenom immunoglobulins and botulism antitoxin), these results should raise awareness for EqPV-H contaminations. Furthermore, other biologicals like live vaccines might harbor infectious EqPV-H, for instance when cell lines used for virus propagation were cultivated with horse serum. Sensitive diagnostic assays should be used for the detection of EqPV-H DNA and a careful risk assessment should be performed when using commercial horse sera in medical and research applications. 

## Figures and Tables

**Table 1:** Primer positions and sequences used for sequencing analysis.

PCR	Primer	Sequence
I	EPVf1	GGGTGGTAAATGCCTTCG
	EPVseqr01	TGGTTGGTGACGCCTGTC
II	EPVseqf01	GACAGGCGTCACCAACCA
	1104R^1^	GGGAATGTCATTGAACGGGAA

^1^ Lu et al. [6].

**Table 2:** General information about the serum samples regarding serum type, land of origin, and the number of individuals.

Serum ID	Serum Type	Origin	Number of Individuals
1	Horse Serum	New Zealand	donor herd
2	Fetal Horse Serum	Mexico	unknown
3	Donor Horse Serum	USA	pooled
4	Horse Serum	USA	donor herd
5	Horse Serum	USA	donor herd
6	Donor Horse Serum	Italy	pooled
7	Horse Serum	France	pooled
8	Donor Horse Serum	Canada	unknown
9	Donor Horse Serum	Chile	unknown
10	Donor Horse Serum	Germany	unknown
11	Donor Horse Serum	Italy	unknown
12	Fetal Horse Serum	Brazil	unknown
13	Donor Horse Serum (heat inactivated)	Chile	unknown
14	Horse Serum	Europe	pooled
15	Donor Horse Serum	Europe	pooled
16	Donor Horse Serum	USA	unknown
17	Horse Serum	New Zealand	unknown
18	Donor Horse Serum	Canada	unknown

**Table 3:** The newly identified specimens were submitted to NCBI and were assigned with respect to the serum ID and the land of origin.

Serum ID	EqPV Sequence Name	NCBI Accession Number
1	Equine Parvovirus H/seq1_NZ_518-1104nt	MK792430
3	Equine Parvovirus H/seq3_USA_518-1104nt	MK792431
4	Equine Parvovirus H/seq4_USA_518-1104nt	MK792432
5	Equine Parvovirus H/seq5_USA_518-1104nt	MK792433
6	Equine Parvovirus H/seq6_IT_518-1104nt	MK792429
11	Equine Parvovirus H/seq11_IT_518-1104nt	MK792434
16	Equine Parvovirus H/seq16_USA_518-1104nt	MK792435
17	Equine Parvovirus H/seq17_NZ_518-1104nt	MK792436
18	Equine Parvovirus H/seq18_CN_518-1104nt	MK792437
1	Equine Parvovirus H/seq1_NZ_20-535nt	MK792438
3	Equine Parvovirus H/seq3_USA_20-535nt	MK792439
4	Equine Parvovirus H/seq4_USA_20-535nt	MK792440
11	Equine Parvovirus H/seq11_IT_20-535nt	MK792441
16	Equine Parvovirus H/seq16_USA_20-535nt	MK792442
17	Equine Parvovirus H/seq17_NZ_20-535nt	MK792443
18	Equine Parvovirus H/seq18_CN_20-535nt	MK792444

## References

[1] Cotmore S.F., Agbandje-McKenna M., Chiorini J.A., Mukha D.V., Pintel D.J., Qiu J., Soderlund-Venermo M., Tattersall P., Tijssen P., Gatherer D. (2014). The family Parvoviridae. Arch. Virol..

[2] Kailasan S., Agbandje-McKenna M., Parrish C.R. (2015). Parvovirus Family Conundrum, What Makes a Killer?. Annu. Rev. Virol..

[3] Palinski R.M., Mitra N., Hause B.M. (2016). Discovery of a novel Parvovirinae virus, porcine parvovirus 7, by metagenomic sequencing of porcine rectal swabs. Virus Genes.

[4] Lau S.K.P., Woo P.C.Y., Yeung H.C., Teng J.L.L., Wu Y., Bai R., Fan R.Y.Y., Chan K.-H., Yuen K.-Y. (2012). Identification and characterization of bocaviruses in cats and dogs reveals a novel feline bocavirus and a novel genetic group of canine bocavirus. J. Gen. Virol..

[5] Divers T.J., Tennant B.C., Kumar A., McDonough S., Cullen J., Bhuva N., Jain K., Chauhan L.S., Scheel T.K.H., Lipkin W.I. (2018). New Parvovirus Associated with Serum Hepatitis in Horses after Inoculation of Common Biological Product. Emerg. Infect. Dis..

[6] Divers T.J., Tomlinson J.E. (2019). Theiler’s disease. Equine Vet Educ..

[7] Lu G., Sun L., Ou J., Xu H., Wu L., Li S. (2018). Identification and genetic characterization of a novel parvovirus associated with serum hepatitis in horses in China. Emerg. Microbes & Infect..

[8] Theiler A. (1918). Acute Liver-Atrophy and Parenchymatous Hepatitis in Horses.

[9] Aleman M., Nieto J.E., Carr E.A., Carlson G.P. (2005). Serum Hepatitis Associated with Commercial Plasma Transfusion in Horses. J. Vet. Intern. Med..

[10] Thomsett L.R. (1971). Acute Hepatic Failure in the Horse. Equine Vet. J..

[11] Chandriani S., Skewes-Cox P., Zhong W., Ganem D.E., Divers T.J., van Blaricum A.J., Tennant B.C., Kistler A.L. (2013). Identification of a previously undescribed divergent virus from the Flaviviridae family in an outbreak of equine serum hepatitis. Proc. Natl. Acad. Sci. USA.

[12] Rose J.A., Immenschuh R.D., Rose E.M. (1974). Serum hepatitis in the horse, Proceedings of the Twentieth Annual Conference of the American Association of Equine Practitioners. Am. Assoc. Equine Pract..

[13] Tomlinson J.E., Kapoor A., Kumar A., Tennant B.C., Laverack M.A., Beard L., Delph K., Davis E., Schott Ii H., Lascola K. (2019). Viral testing of 18 consecutive cases of equine serum hepatitis, A prospective study (2014–2018). J. Vet. Intern. Med..

[14] Tomlinson J.E., Tennant B.C., Struzyna A., Mrad D., Browne N., Whelchel D., Johnson P.J., Jamieson C., Löhr C.V., Bildfell R. (2019). Viral testing of 10 cases of Theiler’s disease and 37 in-contact horses in the absence of equine biologic product administration, A prospective study (2014–2018). J. Vet. Intern. Med..

[15] Burbelo P.D., Ching K.H., Klimavicz C.M., Iadarola M.J. (2009). Antibody profiling by Luciferase Immunoprecipitation Systems (LIPS). J. Vis. Exp..

[16] Burbelo P.D., Dubovi E.J., Simmonds P., Medina J.L., Henriquez J.A., Mishra N., Wagner J., Tokarz R., Cullen J.M., Iadarola M.J. (2012). Serology-enabled discovery of genetically diverse hepaciviruses in a new host. J. Virol..

[17] Pfaender S., Cavalleri J.M.V., Walter S., Doerrbecker J., Campana B., Brown R.J.P., Burbelo P.D., Postel A., Hahn K., Anggakusuma R.N. (2015). Clinical course of infection and viral tissue tropism of hepatitis C virus-like nonprimate hepaciviruses in horses. Hepatology (Baltimore, Md.).

[18] Waddell P.J., Steel M.A. (1997). General time-reversible distances with unequal rates across sites, Mixing gamma and inverse Gaussian distributions with invariant sites. Mol. Phylogenetics Evol..

[19] Felsenstein J. (1981). Evolutionary trees from DNA sequences, A maximum likelihood approach. J. Mol. Evol..

[20] Kumar S., Stecher G., Li M., Knyaz C., Tamura K. (2018). MEGA X, Molecular Evolutionary Genetics Analysis across Computing Platforms. Mol. Biol. Evol..

[21] Stang A., Korn K., Wildner O., Uberla K. (2005). Characterization of virus isolates by particle-associated nucleic acid PCR. J. Clin. Microbiol..

[22] Postel A., Cavalleri J.-M.V., Pfaender S., Walter S., Steinmann E., Fischer N., Feige K., Haas L., Becher P. (2016). Frequent presence of hepaci and pegiviruses in commercial equine serum pools. Vet. Microbiol..

